# A woman with recurrent respiratory tract infections

**DOI:** 10.11604/pamj.2020.37.278.27013

**Published:** 2020-11-26

**Authors:** Petros Ioannou

**Affiliations:** 1 Department of Internal Medicine, University Hospital of Heraklion, Panepistimiou, Iraklio 715 00, Greece

**Keywords:** Kartagener, primary ciliary dyskinesia, dyskinetic cilia syndrome

## Image in medicine

A 76-year-old caucasian woman who has been repeatedly hospitalized with recurrent respiratory tract infections, presented to the emergency department with fever and productive cough during the last 24 hours. Physical examination revealed tachypnea and crackles at the lower right lobe. The chest X-ray revealed an infiltration at the right lower pulmonary field and dextrocardia. Laboratory data revealed leukocytosis and increased C-reactive protein. The electrocardiogram revealed sinus rhythm, right axis deviation, inverted P waves in lead I, dominant R waves in V1 and V2 and dominant S waves in V3-6. Kartagener syndrome is an autosomal recessive disorder also known as “primary ciliary dyskinesia” and “dyskinetic cilia syndrome”. It involves clinical features summarized in a triad including bronchiectasis, chronic sinusitis and dextrocardia/situs inversus. Kartagener syndrome affects equally males and females, with a prevalence of approximately 1 in 10,000-30,000. The underlying cause is impaired ciliary mobility resulting in reduced mucociliary clearance and mucus accumulation causing strong predisposition to recurrent bacterial respiratory tract infections that start during early childhood. Progressively, manifestations such as sinusitis and otitis media increasingly appear along with respiratory tract infections and eventually bronchiectasis develops. Less usual clinical manifestations include telecanthus, infertility in males and subfertility in females. Differential diagnosis includes respiratory allergy, cystic fibrosis, immune disorders and a1-trypsin deficiency. Screening starts with mucociliary clearance measurements but the Kartagener syndrome diagnosis is confirmed with the presence of anatomic abnormalities of the cilia in biopsies of bronchial and nasal mucosa with the use of electron microscopy.

**Figure 1:**
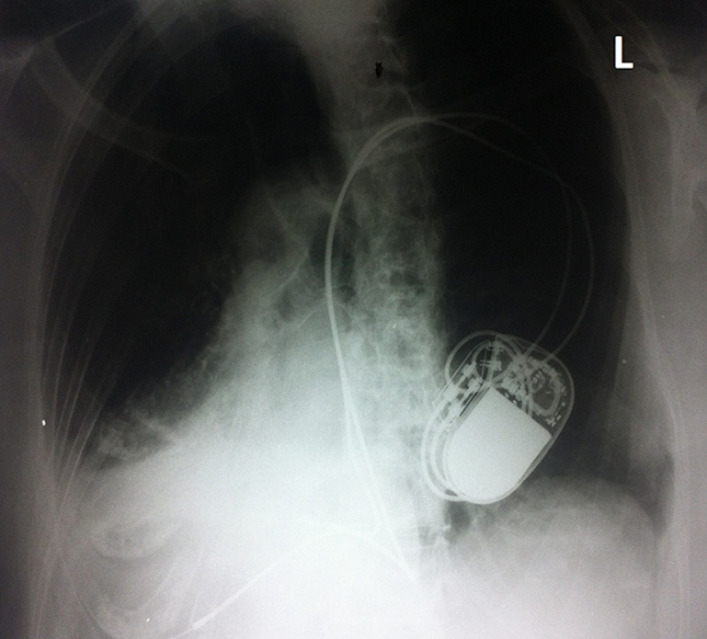
dextrocardia with a permanent implanted pacemaker, right-sided aortic arch suggesting situs inversus; consolidation of the right lower pulmonary field suggesting lower lobe pneumonia

